# Supplementary material for: APOE is a presynaptic protein that accumulates with age and modulates neurotransmitter release
Source: bioRxiv. 2026 Apr 21:2026.04.20.719736. Preprint. [Version 1] doi: 10.64898/2026.04.20.719736 (PMC13131475; doi:10.64898/2026.04.20.719736)
Supplement: Supplement 1 [file NIHPP2026.04.20.719736v1-supplement-1.pdf]

bioRxiv preprint doi: https://doi.org/10.64898/2026.04.20.719736; this version posted April 21, 2026. The copyright holder for this preprint (which was not certified by peer review) is the author/funder, who has granted bioRxiv a license to display the preprint in perpetuity. It is made available under aCC-BY 4.0 International license.

Supplemental Figures and Legends

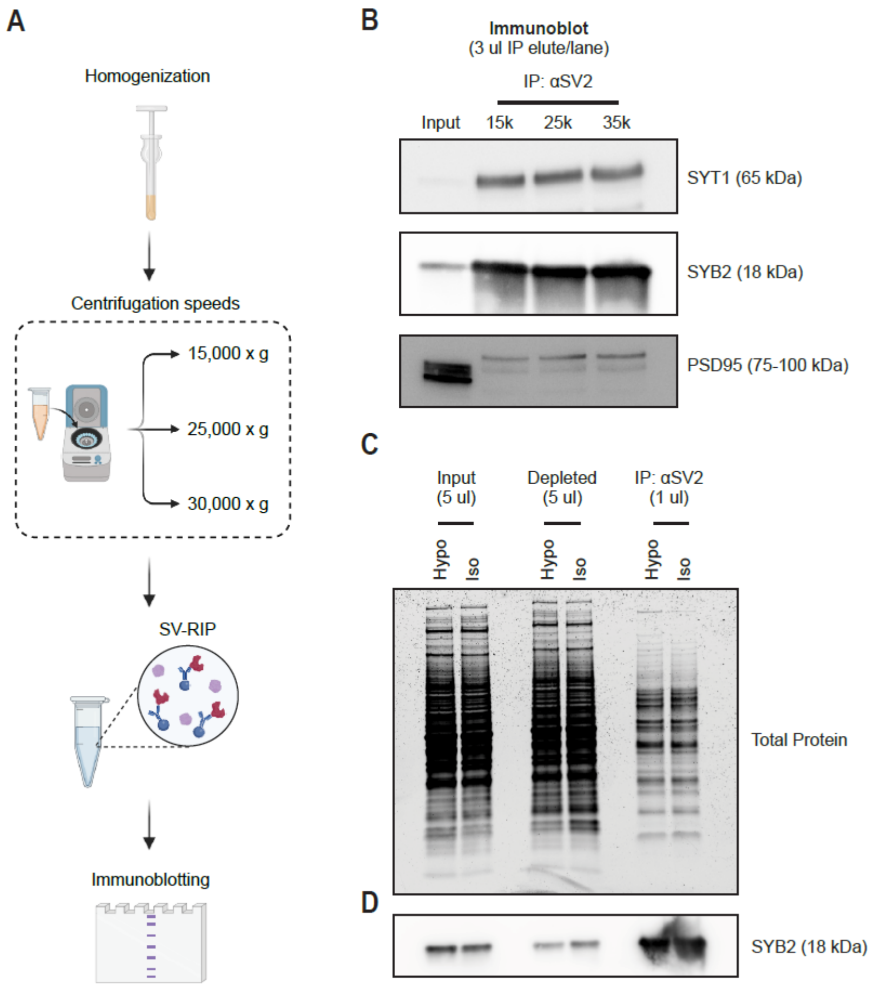

Supplemental Figure 1: SV-RIP validation: Influence of centrifugation speeds on the purity of isolated synaptic vesicles.
A. Experimental workflow for testing different centrifugation speeds during immunoprecipitation. After homogenization, the supernatant was centrifuged at 15,000, 25,000, or 30,000 x g and taken for SV-RIP. Samples were then immunoblotted to assess whether centrifugation speed alters protein composition.
B. Immunoblot analysis of samples centrifuged at 15k, 25k, and 35k x g for synaptic vesicle markers (SYT1 and SYB2) and post-synaptic marker (PSD95) was performed to assess synaptic vesicle enrichment and purity with different centrifugation speeds.
C. Total protein detected for Input, SV-depleted (Depleted), and SV-enriched (IP: αSV2) fractions using hypotonic or isotonic homogenization buffer.
D. Immunoblot analysis of SYB2 for Input, Depleted, and IP: αSV2 fractions using hypotonic or isotonic homogenization buffer.

bioRxiv preprint doi: https://doi.org/10.64898/2026.04.20.719736; this version posted April 21, 2026. The copyright holder for this preprint (which was not certified by peer review) is the author/funder, who has granted bioRxiv a license to display the preprint in perpetuity. It is made available under aCC-BY 4.0 International license.

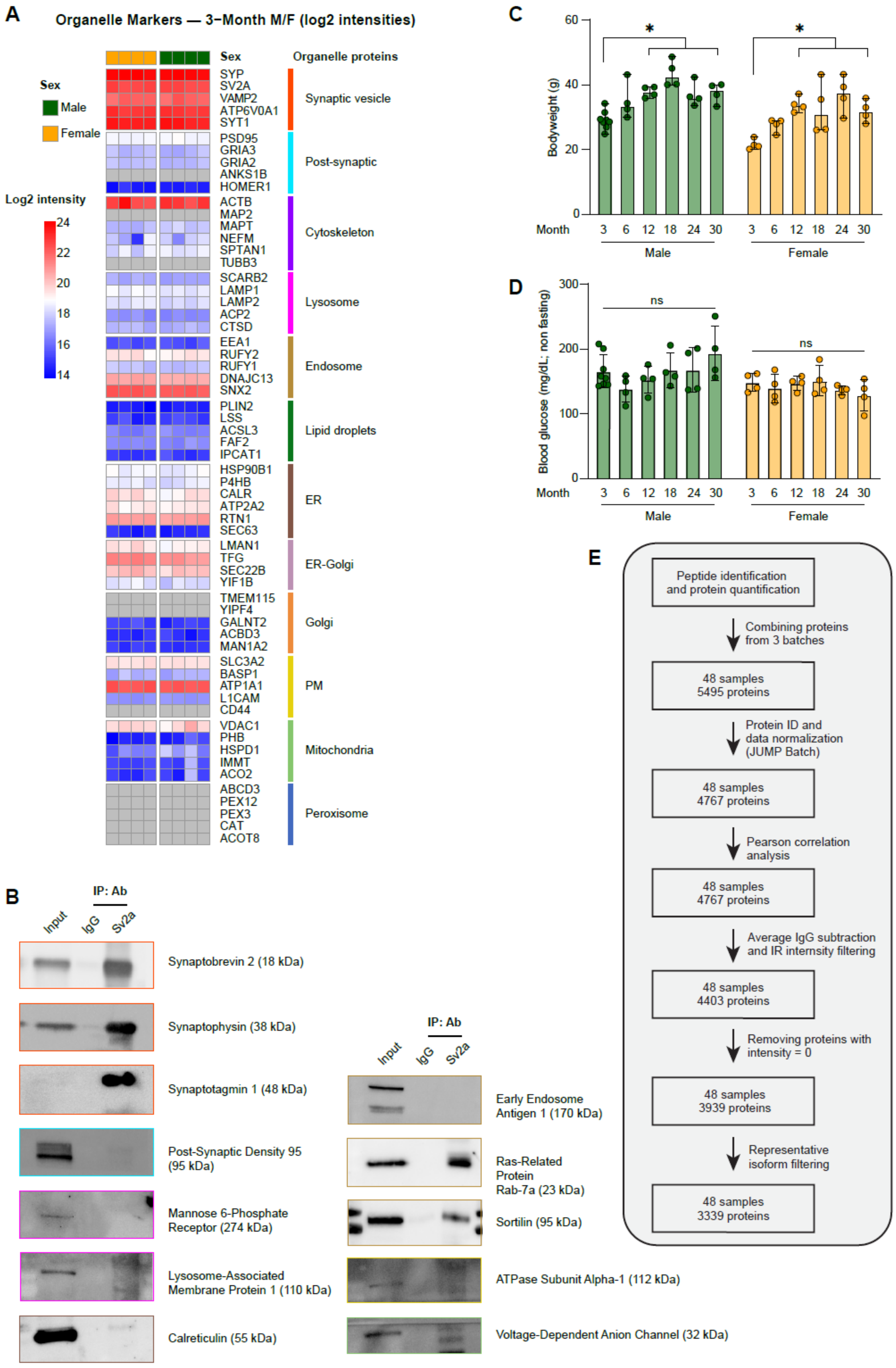

bioRxiv preprint doi: https://doi.org/10.64898/2026.04.20.719736; this version posted April 21, 2026. The copyright holder for this preprint (which was not certified by peer review) is the author/funder, who has granted bioRxiv a license to display the preprint in perpetuity. It is made available under aCC-BY 4.0 International license.

Supplemental Figure 2: Additional validation of SV-RIP enrichment and mouse biomarker analysis across groups.

A. Log2 intensity of organelle markers from 3-month male and female samples detected from TMT proteomics.

B. Organelle markers examined by western blot from Input, IgG IP, and SV2a IP.

C. Body weight (grams) at collection for male and female C57BL6/J mice. Data are displayed as median with 95% confidence intervals and analyzed by 2-way ANOVA with Dunnett's multiple comparisons test (* $p<0.05$).

D. Non-fasting blood glucose levels (mg/dL) at collection for male and female C57BL6/J mice. Data are displayed as median with 95% confidence intervals and analyzed by 2-way ANOVA with Dunnett's multiple comparisons test.

E. Proteomic data analysis workflow. Using the St. Jude JUMPm software, peptides were identified and quantified from 48 samples, and proteins detected in all three batches combined (5495 proteins). Protein IDs were assigned and data normalized using JUMP Batch (4767 proteins), and Pearson correlation analysis was performed. The average IgG intensity was subtracted for each protein, and those not detected in the Internal Reference (IR) samples removed (4404 proteins). Proteins with an intensity of 0 in any sample were removed (3939 proteins). Finally, proteins were filtered to retain one isoform, and 3339 proteins were taken for downstream statistical analysis.

St. Jude - Confidential

bioRxiv preprint doi: https://doi.org/10.64898/2026.04.20.719736; this version posted April 21, 2026. The copyright holder for this preprint (which was not certified by peer review) is the author/funder, who has granted bioRxiv a license to display the preprint in perpetuity. It is made available under aCC-BY 4.0 International license.

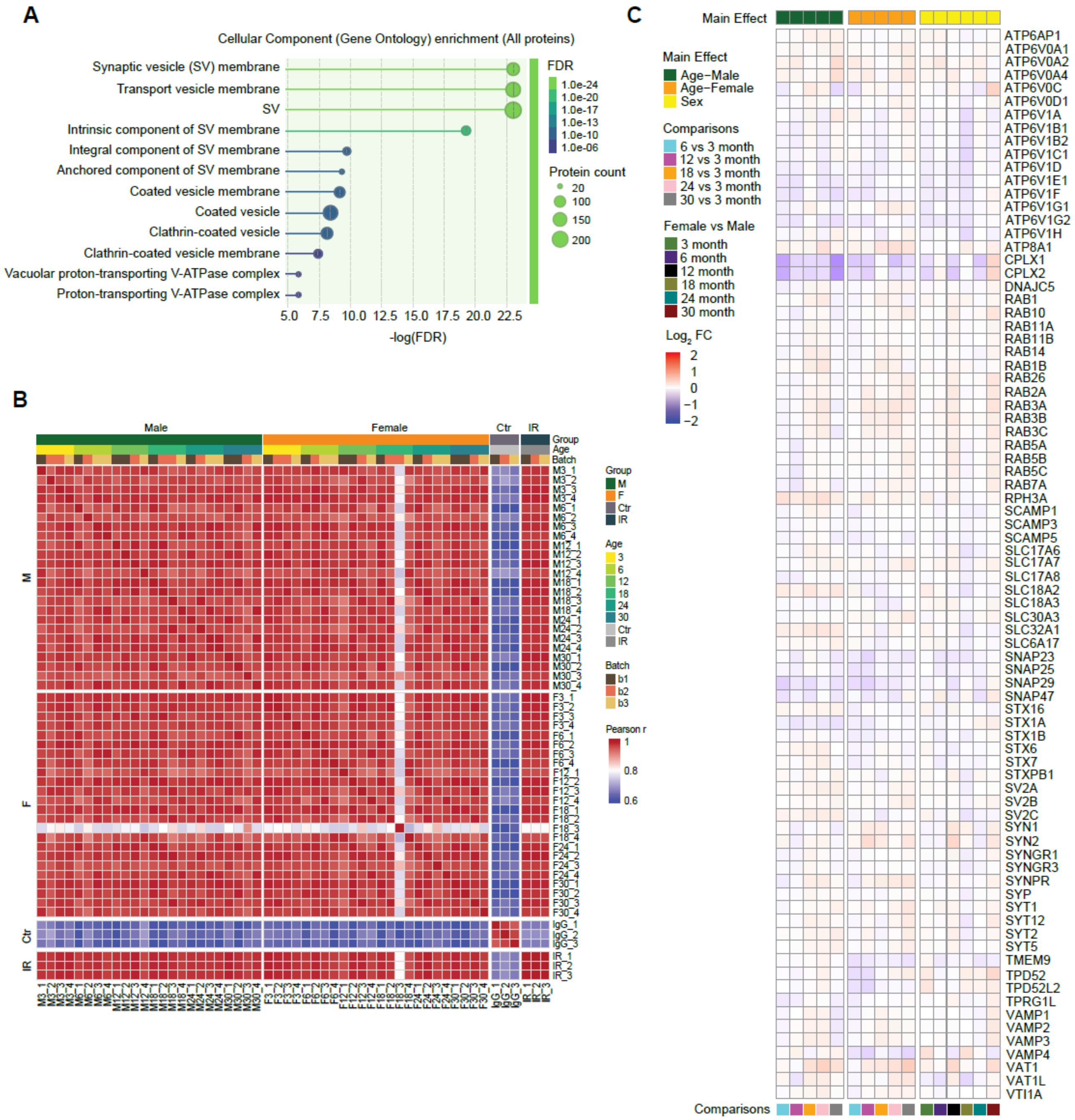

Supplemental Figure 3: Additional proteomic data analysis

A. Cellular component gene ontology enrichment analysis of all detected proteins shows enrichment of synaptic vesicle components.

B. Pearson correlation plot of 4764 normalized proteins from Male, Female, IgG Control (Ctr), and Internal Reference (IR) samples.

C. Fold change of synaptic vesicle proteins for age in males (6 vs 3 month, 12 vs 3 month, 18 vs 3 month, 24 vs 3 month, and 30 vs 3 month), age in females (6 vs 3 month, 12 vs 3 month, 18 vs 3 month, 24 vs 3 month, and 30 vs 3 month), and sex (Female vs Male 3 month, 6 month, 12 month, 18 month, 24 month, and 30 month).

bioRxiv preprint doi: https://doi.org/10.64898/2026.04.20.719736; this version posted April 21, 2026. The copyright holder for this preprint (which was not certified by peer review) is the author/funder, who has granted bioRxiv a license to display the preprint in perpetuity. It is made available under aCC-BY 4.0 International license.

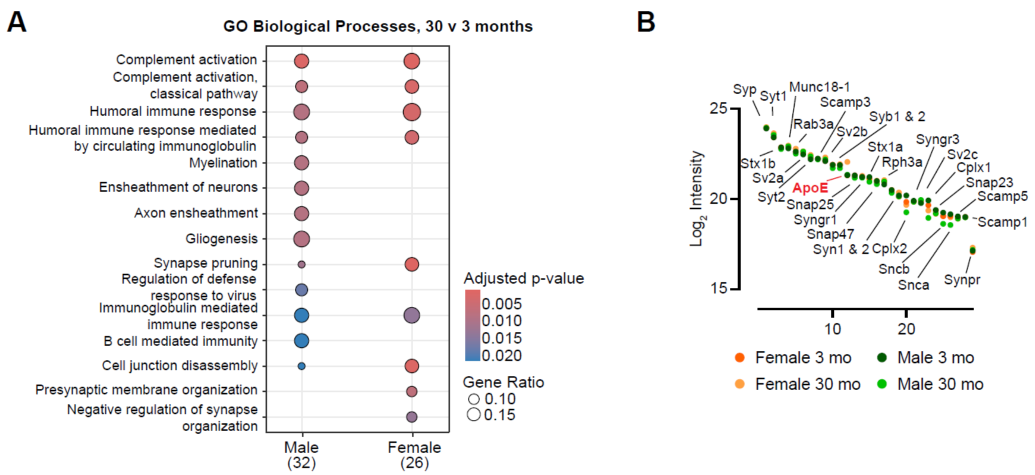

Supplemental Figure 4: Additional analysis of proteomic changes between young and old SVs from male and females.

A. Top GO Biological Processes for proteins significantly altered between 3 and 30 months for male and female samples.

B. Rank ordered log2 intensity of Core SV proteins from 3- and 30-month-old males and females as shown in Main Figure 2E, now with levels of APOE added to chart demonstrating APOE is an abundant SV protein in males and females.

bioRxiv preprint doi: https://doi.org/10.64898/2026.04.20.719736; this version posted April 21, 2026. The copyright holder for this preprint (which was not certified by peer review) is the author/funder, who has granted bioRxiv a license to display the preprint in perpetuity. It is made available under aCC-BY 4.0 International license.

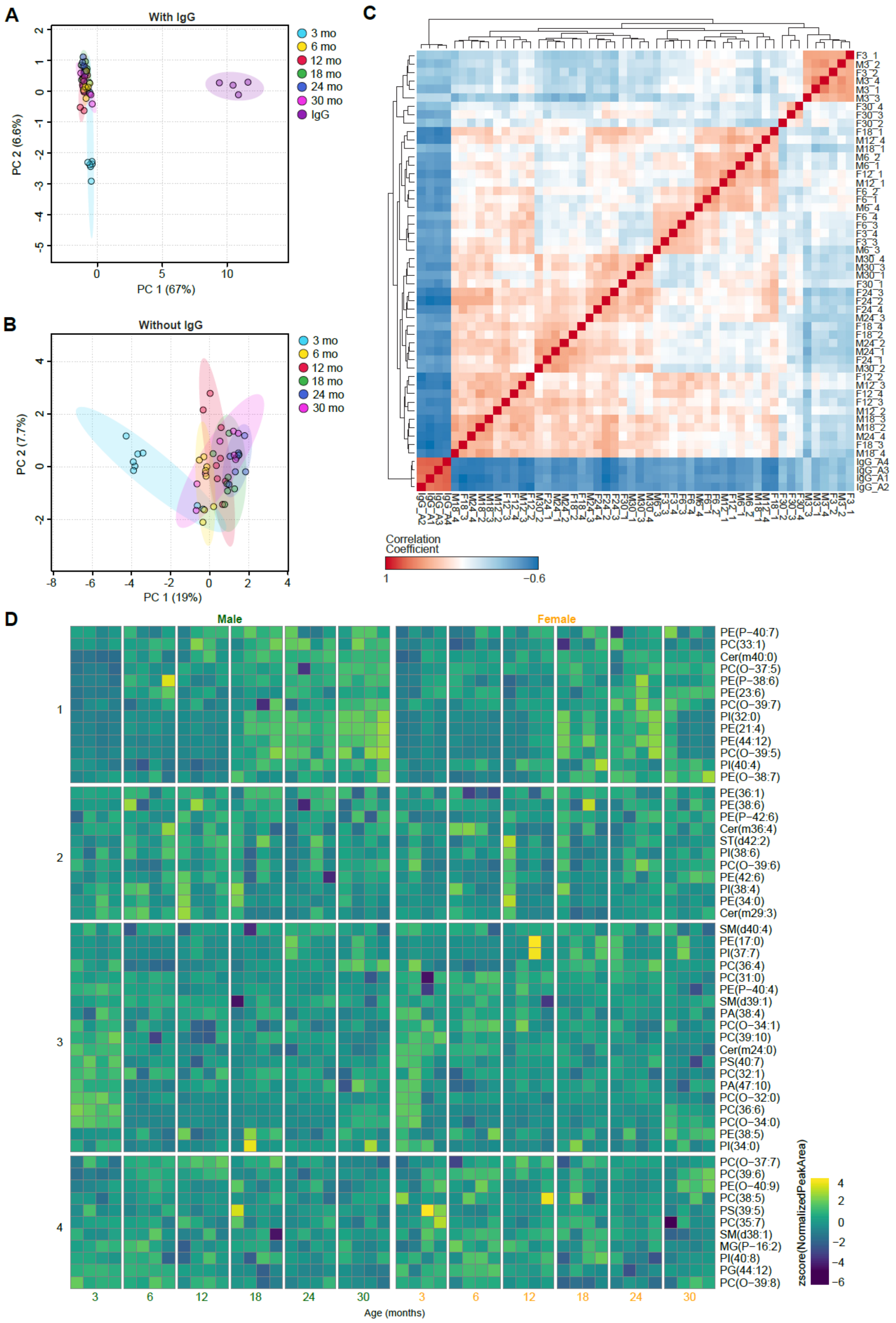

bioRxiv preprint doi: https://doi.org/10.64898/2026.04.20.719736; this version posted April 21, 2026. The copyright holder for this preprint (which was not certified by peer review) is the author/funder, who has granted bioRxiv a license to display the preprint in perpetuity. It is made available under aCC-BY 4.0 International license.

Supplemental Figure 5: Lipidomic quality control and further analysis

A. Principal component analysis of detected phospholipids at all ages (sex combined) for IgG and SV samples. IgG samples separate from SV samples in the first component.

B. Principal component analysis of detected phospholipids at all ages (sex combined) for SV samples only. 3 month samples separate from other ages in the first component.

C. Correlation plot of phospholipids detected from untargeted lipidomics for all IgG and SV samples. IgG phospholipids are highly correlated, and distinct from SV phospholipids.

D. Phospholipids with a significant Age and Sex interaction (FDR ≤0.05). Values displayed are z-score of normalized peak area for each significantly altered phospholipid from male and female synaptic vesicles at 3-, 6-, 12-, 18-, 24-, and 30-months. Data were analyzed by limma linear regression with Benjamini-Hochberg multiple testing correction and separated into 4 k-means clusters.

St. Jude - Confidential

bioRxiv preprint doi: https://doi.org/10.64898/2026.04.20.719736; this version posted April 21, 2026. The copyright holder for this preprint (which was not certified by peer review) is the author/funder, who has granted bioRxiv a license to display the preprint in perpetuity. It is made available under aCC-BY 4.0 International license.

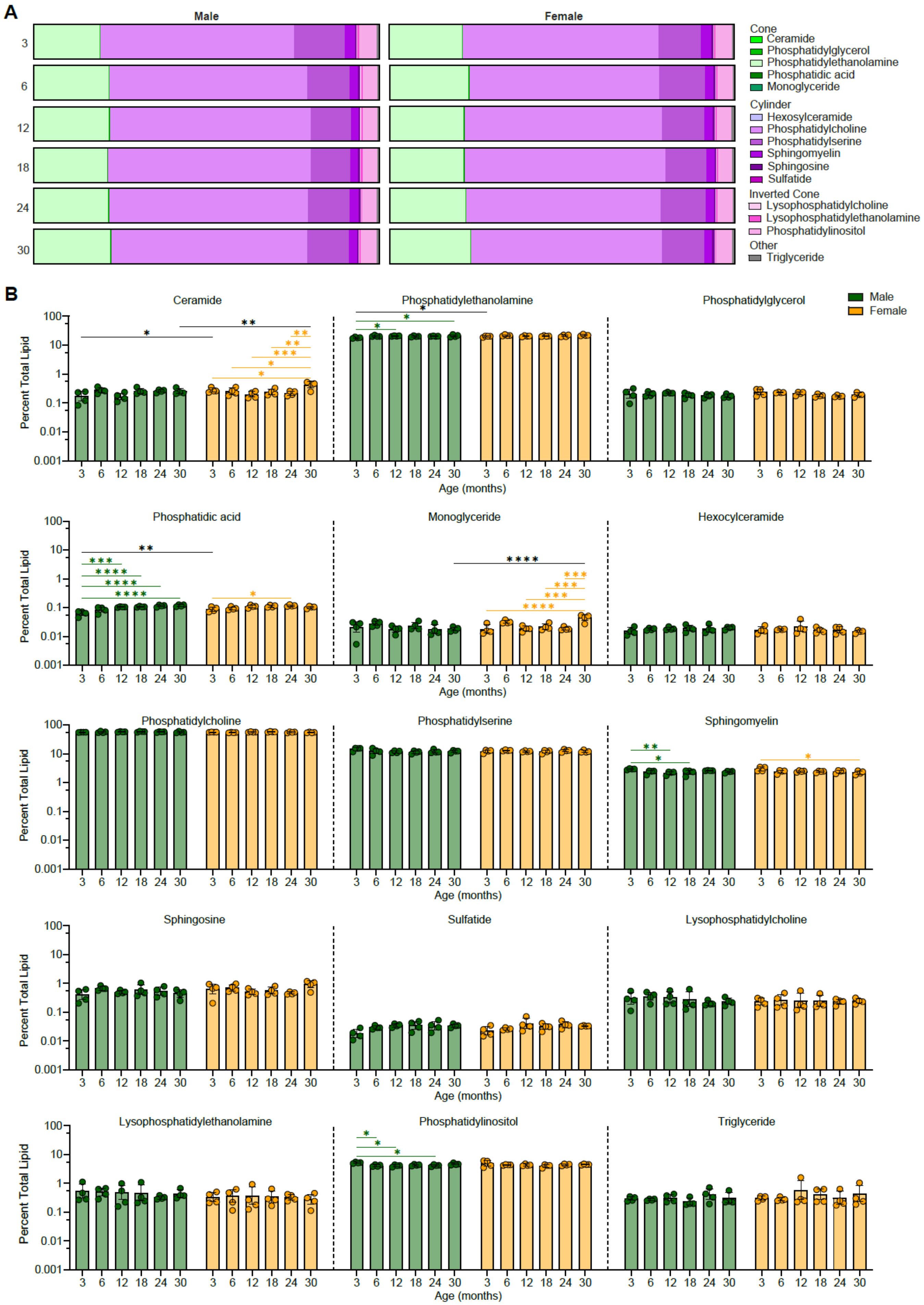

bioRxiv preprint doi: https://doi.org/10.64898/2026.04.20.719736; this version posted April 21, 2026. The copyright holder for this preprint (which was not certified by peer review) is the author/funder, who has granted bioRxiv a license to display the preprint in perpetuity. It is made available under aCC-BY 4.0 International license.

Supplemental Figure 6: Phospholipid class analysis

A. Average percent total lipid composition by lipid class for male and female synaptic vesicles at 3-, 6-, 12-, 18-, 24-, and 30-months.

B. Percent total composition of individual lipid classes (ceramide, phosphatidylethanolamine, phosphatidylglycerol, phosphatidic acid, monoglyceride, hexocylceramide, phosphatidylcholine, phosphatidylserine, sphingomyelin, sphingosine, sulfatide, lysophosphatidylcholine, lysophosphatidylethanolamine, phosphatidylinositol, and triglyceride) for male and female synaptic vesicles at 3-, 6-, 12-, 18-, 24-, and 30-months. Data are displayed as means with standard deviation and analyzed by 2-way ANOVA with Tukey's multiple comparisons test (* $p<0.05$, ** $p<0.01$, *** $p<0.001$, **** $p<0.0001$).

bioRxiv preprint doi: https://doi.org/10.64898/2026.04.20.719736; this version posted April 21, 2026. The copyright holder for this preprint (which was not certified by peer review) is the author/funder, who has granted bioRxiv a license to display the preprint in perpetuity. It is made available under aCC-BY 4.0 International license.

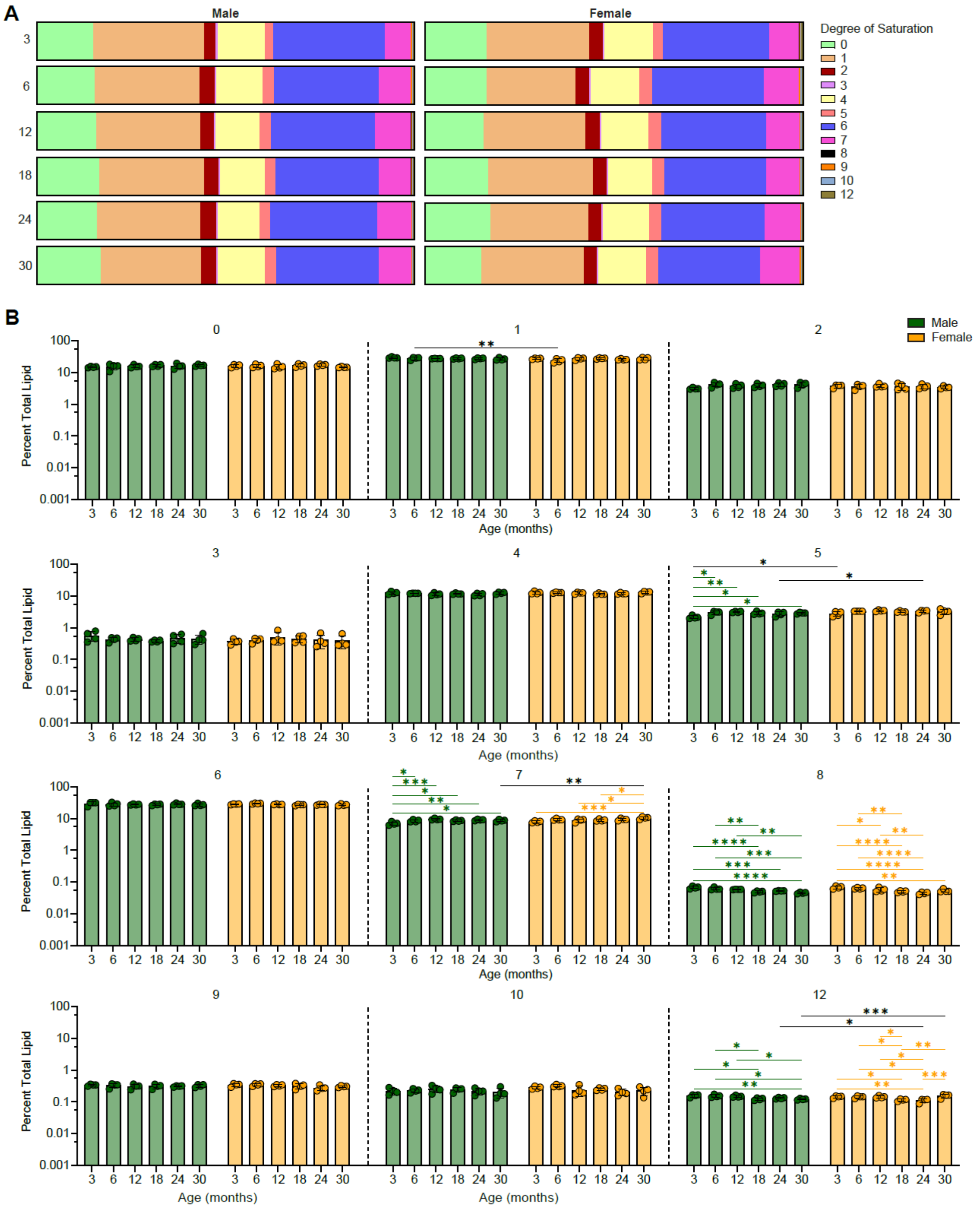

Supplemental Figure 7: Phospholipid sidechain analysis

A. Average percent total lipid composition by lipid saturation for male and female synaptic vesicles at 3-, 6-, 12-, 18-, 24-, and 30-months.

B. Percent total lipid composition by degree of saturation (0, 1, 2, 3, 4, 5, 6, 7, 8, 9, 10, 12 double bonds) for male and female synaptic vesicles at 3-, 6-, 12-, 18-, 24-, and 30-months. Data are displayed as means with standard deviation and analyzed by 2-way ANOVA with Tukey's multiple comparisons test (* p<0.05, ** p<0.01, *** p<0.001, **** p<0.0001).

bioRxiv preprint doi: https://doi.org/10.64898/2026.04.20.719736; this version posted April 21, 2026. The copyright holder for this preprint (which was not certified by peer review) is the author/funder, who has granted bioRxiv a license to display the preprint in perpetuity. It is made available under aCC-BY 4.0 International license.

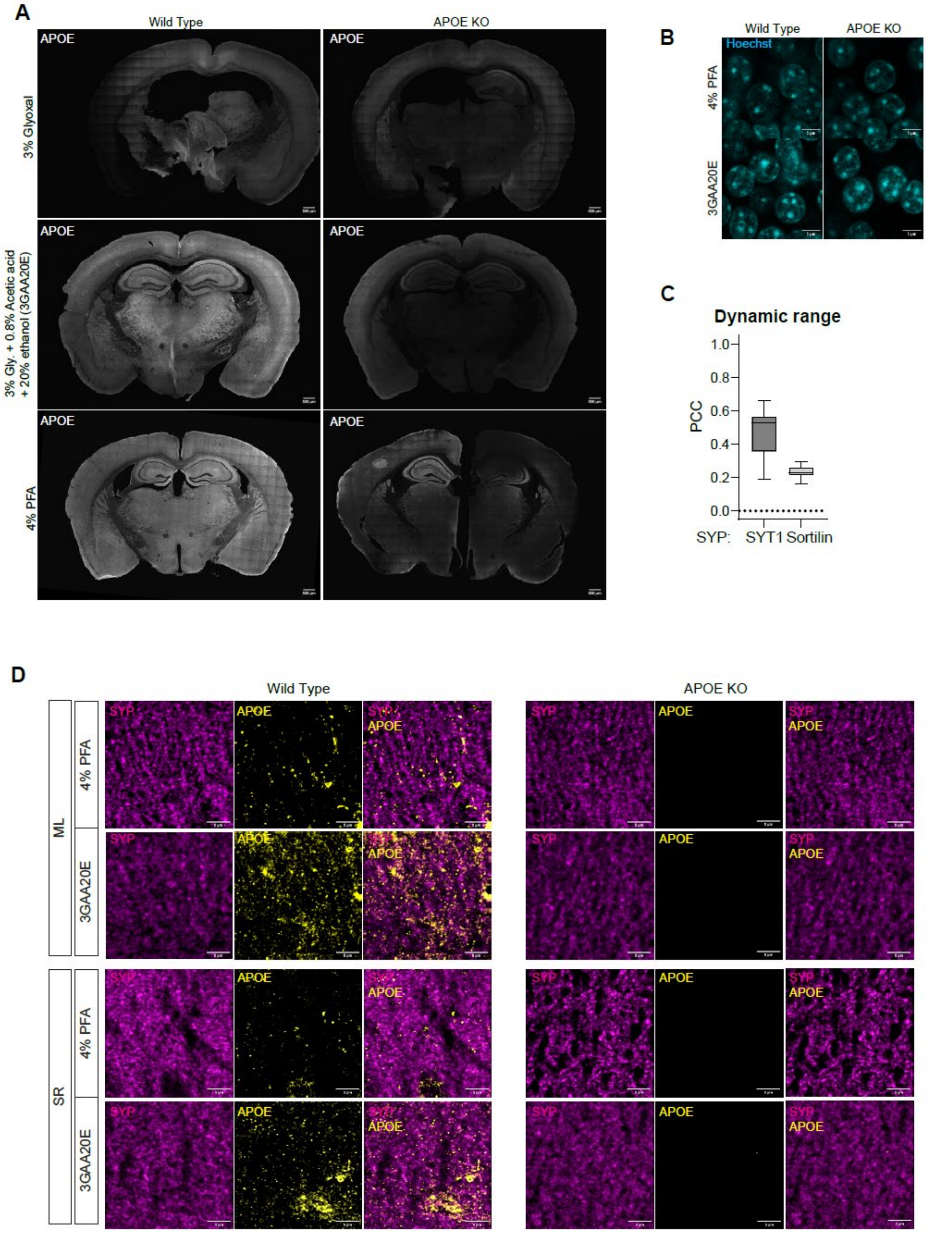

1460

bioRxiv preprint doi: https://doi.org/10.64898/2026.04.20.719736; this version posted April 21, 2026. The copyright holder for this preprint (which was not certified by peer review) is the author/funder, who has granted bioRxiv a license to display the preprint in perpetuity. It is made available under aCC-BY 4.0 International license.

Supplemental Figure 8: Brain tissue fixation optimization and comparison

A. Whole-section immunohistochemical comparison of APOE labeling in wild-type (left column) and APOE knockout (KO; right column) mouse brains fixed using three different fixatives including 3% glyoxal (top row), 3GAA20E (middle row), and 4% PFA (bottom row).

B. Nuclear morphology and size appear comparable between two fixatives. Hoechst staining of wild-type (left column) and APOE KO (right column) mouse brains fixed with 4% PFA (top row) or 3GAA20E (bottom row).

C. Dynamic range assessment of the colocalization analysis using Pearson's correlation coefficient (PCC). Positive (SYP-SYT1) and negative (SYP-Sortilin) marker pairs were stained to demonstrate that the analysis method reliably distinguishes true synaptic colocalization from non-colocalizing signals.

D. Comparison of APOE immunostaining quality in the molecular layer (ML) and stratum radiatum (SR) of APOE wild-type and knockout mouse hippocampi fixed with 4% PFA (top row) or 3GAA20E (bottom row). Anti-APOE labeling (yellow) in the KO tissue serves as a negative control, revealing reduced background fluorescence and improved signal-to-noise with 3GAA20E fixation relative to PFA. The anti-SYP staining (magenta) highlights synaptic structures present in both conditions.

St. Jude - Confidential

bioRxiv preprint doi: https://doi.org/10.64898/2026.04.20.719736; this version posted April 21, 2026. The copyright holder for this preprint (which was not certified by peer review) is the author/funder, who has granted bioRxiv a license to display the preprint in perpetuity. It is made available under aCC-BY 4.0 International license.

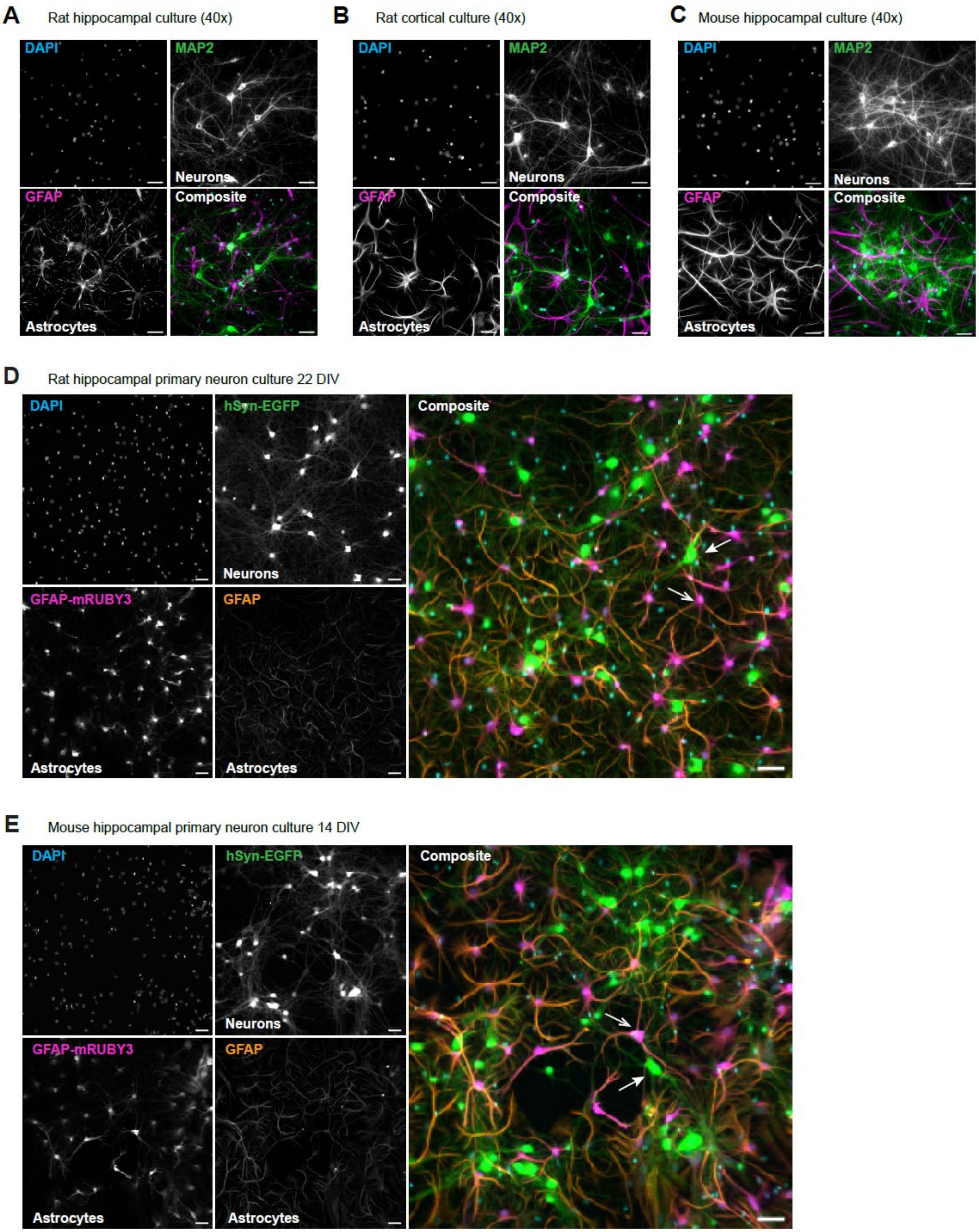

1476

1477 **Supplemental Figure 9: Validation of mixed neuron-astrocyte co-cultures**

bioRxiv preprint doi: https://doi.org/10.64898/2026.04.20.719736; this version posted April 21, 2026. The copyright holder for this preprint (which was not certified by peer review) is the author/funder, who has granted bioRxiv a license to display the preprint in perpetuity. It is made available under aCC-BY 4.0 International license.

A. Representative immunofluorescence image demonstrating the presence of both neurons and astrocytes in DIV21 rat hippocampal cultures. Neurons are visualized with MAP2 (green), astrocytes with GFAP (magenta) and nuclei with DAPI (blue).
B. Representative immunofluorescence visualization confirming the presence of neurons and astrocytes in DIV21 rat cortical cultures. Neurons are marked by MAP2 (green), astrocytes by GFAP (magenta), and nuclei by DAPI (blue).
C. Representative immunofluorescence image illustrating neuronal and astrocytic populations in DIV14 mouse hippocampal culture labeled with MAP2 and GFAP. Stained with anti-MAP2 (green), anti-GFAP (magenta), and DAPI (blue).
D. Lentiviral delivery of a *GFAP: cytosolic mRUBY3* construct at high titer drives reporter expression selectively in astrocytes in DIV22 rat hippocampal culture. Neurons exhibit robust hSyn-EGFP expression (green), while astrocytes display strong GFAP: cytosolic mRUBY3 fluorescence (orange).
E. In DIV14 mouse hippocampal cultures, high titer of hSyn: cytosolic msGFP and GFAP: cytosolic mRUBY3 lentiviruses drive cell-type-specific labeling, with mRUBY3 restricted to GFAP-positive astrocytes and absent from neurons, validating promoter specificity.

bioRxiv preprint doi: https://doi.org/10.64898/2026.04.20.719736; this version posted April 21, 2026. The copyright holder for this preprint (which was not certified by peer review) is the author/funder, who has granted bioRxiv a license to display the preprint in perpetuity. It is made available under aCC-BY 4.0 International license.

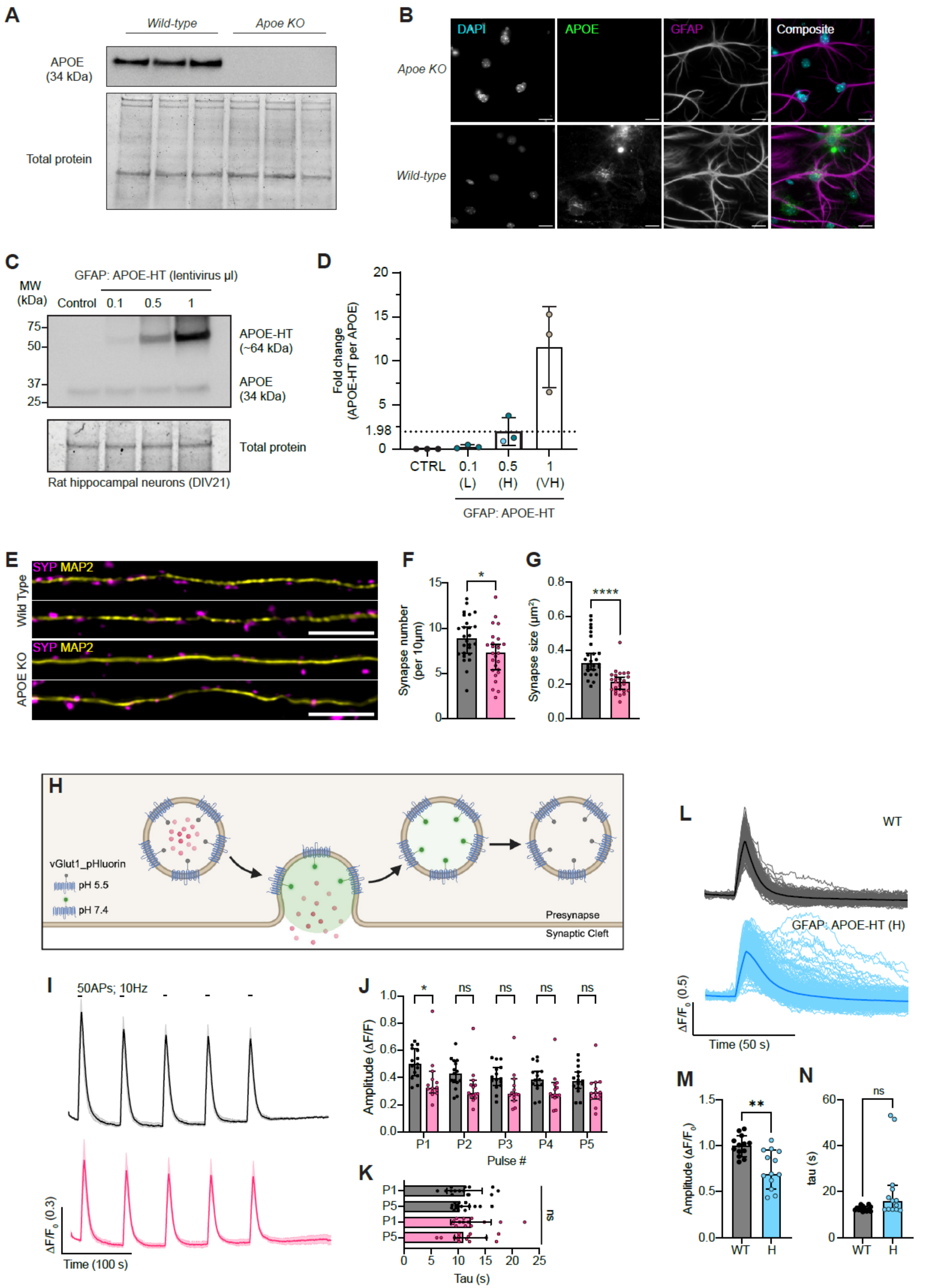

St. Jude - Confidential

bioRxiv preprint doi: https://doi.org/10.64898/2026.04.20.719736; this version posted April 21, 2026. The copyright holder for this preprint (which was not certified by peer review) is the author/funder, who has granted bioRxiv a license to display the preprint in perpetuity. It is made available under aCC-BY 4.0 International license.

Supplemental Figure 10: Modulation of APOE protein levels and synaptic characterization

A. Representative immunoblot from 3 separate DIV14 hippocampal cultures from either wild type or APOE KO mice probed for APOE.

B. Representative immunofluorescence image localizing APOE in wild-type and APOE KO DIV14 mouse hippocampal neuron astrocyte cocultures. Stained with DAPI (blue), anti APOE (green), and anti GFAP (magenta).

C. Representative immunoblot from WT rat hippocampal DIV21 neuron astrocyte cocultures from control, and GFAP: APOE-HaloTag transduced cultures using 0.1 µl lentivirus (Low; L), 0.5 µl lentivirus (High; H), or 1.0 µl lentivirus (Very High; VH).

D. Quantification of triplicate repeat immunoblots as shown in (F).

E. Primary mouse hippocampal cocultures fixed and stained with anti MAP2 (yellow) and anti SYP (magenta). MAP2 regions straightened using ImageJ. Scale bar 5 microns.

F. Synapse number (SYP punctae) per 10 microns. Values are median ± 95% confidence interval, n = 26 dendrite stretches per condition, Mann-Whitney two-tailed test, $p < 0.05$.

G. Synapse size (SYP area) between WT and APOE KO cocultures. Values are median ± 95% confidence interval, n = 26 dendrite stretches per condition, Mann-Whitney two-tailed test, $p < 0.0001$.

H. Schematic of vGlut1-pHluorin during exocytosis and endocytosis.

I. Average trace of vGlut1-pHluorin with 50 action potentials; 10Hz train stimulus repeated 5 times with 1 minute rest in between trains; traces are mean± 95% confidence interval.

J. Amplitude of evoked pHluorin events ($\Delta F/F_0$) from WT and APOE KO hippocampal cultures. Values are median ± 95% confidence interval, n = 14-15 neurons per condition. Two-way ANOVA with Sidak's multiple comparisons test (* $p<0.05$)

K. Time constant (tau) of the decay of first and fifth pHluorin peaks for WT and APOE KO hippocampal cultures. Values are median ± 95% confidence interval, n = 14-15 neurons per condition. One-way ANOVA with Dunnett's multiple comparisons test.

L. Representative traces from a single field of view (FOV) of Rat hippocampal neuron astrocyte cocultures expressing vGlut1-pHluorin and stimulated with a high frequency train of 100 action potentials at 20 Hz. WT in black and GFAP: APOE-HT OE (0.5 µl, High) in blue/light blue. Individual traces in light colors with FOV average in dark.

M. Peak amplitude ($\Delta F/F_0$) of vGlut1-pHluorin from WT and GFAP: APOE-HT OE (0.5 µl, High). Values are median ± 95% confidence interval, n = 13 FOV per condition, Mann-Whitney two-tailed test, $p < 0.005$.

N. Time constant (tau) of vGlut1-pHluorin decay after stimulation for WT and GFAP: APOE-HT OE (0.5 µl, High). Values are median ± 95% confidence interval, n = 13 FOV per condition, Mann-Whitney two-tailed test, ns = 0.0501.
